# Different phases of aging in mouse old skeletal muscle

**DOI:** 10.18632/aging.203812

**Published:** 2022-01-11

**Authors:** Yong-Kook Kang, Byungkuk Min, Jaemin Eom, Jung Sun Park

**Affiliations:** 1 Development and Differentiation Research Center, Korea Research Institute of Bioscience Biotechnology (KRIBB), Yuseong-Gu, Daejeon 34141, South Korea; 2 Department of Functional Genomics, Korea University of Science and Technology (UST), Yuseong-Gu, Daejeon 34113, South Korea

**Keywords:** skeletal muscle, early and late phase aging, transcriptome, young-old, old-old

## Abstract

With a graying population and increasing longevity, it is essential to identify life transition in later years and discern heterogeneity among older people. Subclassifying the elderly population to inspect the subdivisions for pathophysiological differences is particularly important for the investigation of age-related illnesses. For this purpose, using 24- and 28-month-old mice to represent the “young-old” and “old-old”, respectively, we compared their skeletal muscle transcriptomes and found each in a distinct stage: early/gradual (E-aging) and late/accelerated aging phase (L-aging). Principal component analysis showed that the old-old transcriptomes were largely disengaged from the forward transcriptomic trajectory generated in the younger-aged group, indicating a substantial change in gene expression profiles during L-aging. By calculating the transcriptomic distance, it was found that the 28-month group was closer to the two-month group than to the 24-month group. The divergence rate per month for the transcriptomes was the highest in L-aging, twice as fast as the rate in E-aging. Indeed, many of the L-aging genes were significantly altered in transcription, although the changes did not seem random but rather coordinated in a variety of functional gene sets. Of 2,707 genes transcriptionally altered during E-aging, two-thirds were also significantly changed during L-aging, to either downturning or upturning way. The downturn genes were related to mitochondrial function and translational gene sets, while the upturn genes were linked to inflammation-associated gene sets. Our results provide a transcriptomic muscle signature that distinguishes old-old mice from young-old mice. This can help to methodically examine muscle disorders in the elderly.

## INTRODUCTION

As demographic aging continues, the population structure is shifting to an uncharted phase in which the “super elderly” (80 years and older) outnumber the “elderly” (60 years and older). Older adults are at risk of developing age-associated diseases, so this transition heavily weighs on government finances. Thus, maintaining adult health while aging is important, both for individual quality of life as well as costs to healthcare systems [1].

With a graying population and increasing longevity, it is important to identify life transitions in later years and recognize heterogeneity among older people [2]. The term “late life” is broadly defined by encompassing a heterogeneous group of adults of 65 years and older; hence, it is further classified into “young-old” and “old-old” groups [3, 4] in the hope of identifying the group with a distinct vulnerability to certain chronic diseases and mental illnesses. Supportively, several studies have discerned a comprehensive difference across physical, cognitive, and psychosocial domains between the young-old (aged 60 – 74 years) and old-old (aged 75 years and older) groups [2, 5, 6]. A similar distinction may exist for physiological and pathological domains, such as chronic illnesses (cardiovascular disease, cancer, chronic respiratory diseases, and diabetes, among others) and the deterioration of skeletal muscle and cognitive function [7]. In reality, these age-related illnesses vary markedly and can, with age, take the shape of a comorbidity, which is the co-existence of two or more diseases [8]. For instance, only 30% of adults aged 45 – 64 years have at least two chronic conditions, whereas 65% of those aged 65 – 84 years and approximately 80% of those aged 85 years and older have the same conditions [9]. Therefore, to investigate these age-associated diseases, it may be beneficial to divide the elderly into groups and inspect the resultant subgroups separately for pathophysiological differences, and other deteriorations or weaknesses.

In the case of mice, those ranging from 18 to 24 months-of-age, which is comparable to humans of 56 – 69 years-of-age, fulfil the requirements of “young-old” age, whereas mice aged 26 months and older can be considered as “old-old” [10]. It is notable that 22 – 24 months of age is when morphological changes consistent with human sarcopenia [11] commence in mice and rats [12, 13]. This is the period skeletal muscle mass and grip strength decline progressively with age, exhibiting prominent changes at 24-28 months of age, while whole–body mass and lean mass were relatively stable or only marginally declined [13]. Another significant distinction between the young-old and old-old groups is survivorship; 24- and 28-month-old mice exhibit 85% and 50% survival rates, respectively [14, 15], or less depending on the strain and sex (Strain survival information, https://www.nia.nih.gov). Based on this rapid declines in muscle mass and survivorship with age, we assumed that aging accelerates in “late life” in a manner different from that in the slow aging mode before then. In addition to the increased morbidity and accelerated aging, we recently noticed that skeletal muscle in old-old mice, but not in young-old mice, underwent DNA demethylation particularly over genomic retroelements, and as a consequence, a large number of genomic retroelement copies acquire the competence for transcription [16]. Similarly, the existence of other unexplored molecular and physiological traits that distinguish old-old mice from young-old mice, is also conceivable.

Recently, there were reports of bulk [17] and single-cell RNA-seq analysis [18] on aging hallmarks across the organs and age in mice, but the global scale in these studies has benefit for understanding overall picture of aging but is not greatly helpful to study in detail aging events particularly in skeletal muscle and in “later life” if not thoroughly re-analyzed. We examined the transcriptomes of skeletal muscle sampled from 24- and 28-month-old mice as the young-old and old-old groups, respectively, along with 2-, 10-, and 18-month-old mice representing young and midlife controls. By focusing on the genes that deviated from the normal expression profiles in the late aging phase, our study provided insight into the transcriptomic features of the skeletal muscle of old-old mice compared to that of young-old mice. Furthermore, we investigated whether there are any genes in which the expression shifts are coordinately regulated in accordance with the transition in “late life”; thereby, providing a thorough impact assessment of the late aging phase.

## RESULTS

### Comprehensive gene expression changes during late phase of aging

**Figure 1:**
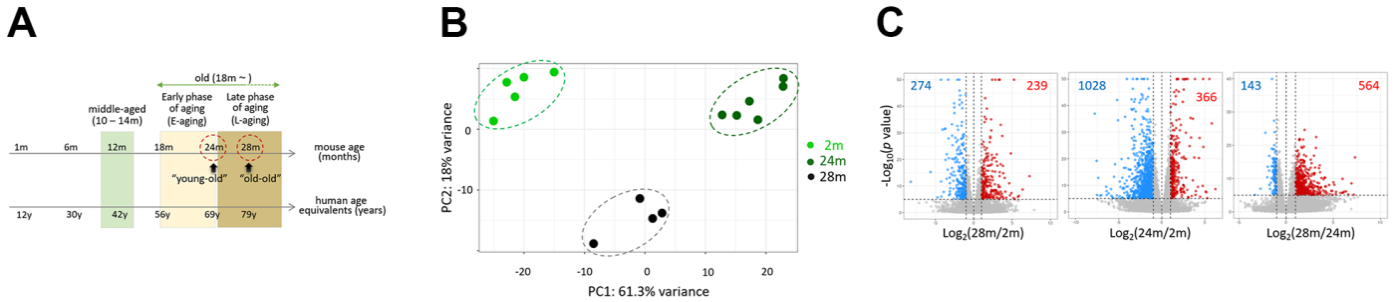
**Comparison of skeletal muscle transcriptomes of 2-, 24-, and 28-month-old mice.** (**A**) Representative age ranges for mature life history stages in C57BL/6J mice are shown, together with human age equivalents: 10 to 14 months-of-age as middle age and 18 – 24 months as early phase of aging (E-aging) [10]. We added the life phase of 25 – 28 months or older as late phase of aging (L-aging); note that we substituted the “old” definition in the online article [10] with E-aging. We named 24 and 28 months-of-age as “young-old” and “old-old”. Aging phases are shaded in different colors. (**B**) Principal component analysis (PCA). Transcriptomes of different age groups are marked as dots and lines with different colors. 2m, 24m, and 28m stand for 2-, 24-, and 28-month-old mice, respectively. (**C**) Volcano plots for comparison of gene expression between 2m versus 28m (left), 2m versus 24m (middle), and 24m versus 28m (right). Differentially expressed genes (log_2_ (fold change > 1.0 and p < 1 × 10^–5^) are colored.

We obtained RNA sequencing data from the skeletal muscle of 2- (2m; n = 5), 24- (24m; n = 6), and 28-month-old mice (28m; n = 4) to compare their transcriptomes. We regarded 24 and 28 months-of-age as young-old and old-old and provisionally designated the corresponding periods as the early aging (E-aging) and late aging phase (L-aging), respectively (Figure 1A and Introduction for the rationale). Principal component analysis (PCA) result showed that the transcriptomes of young, young-old, and old-old age groups clearly diverged from each other (Figure 1B). In total, 707 differentially expressed genes (DEGs; fold-change > 2 and *P* < 1 × 10^–5^; Supplementary File 1) were identified in the comparison of 28m and 24m samples. This DEG number was considerable for a short period of only four months, and comparable to the 1,394 DEGs detected in the 24m-versus-2m comparison (Figure 1C and Supplementary File 1). This indicated that global gene expression changes occur during L-aging. We confirmed differential expressions of the identified DEGs in the young-old and old-old muscle samples through a quantitative real-time PCR (Supplementary Figure 1).

### Some cellular processes were disturbed in a coordinated and L-aging-specific fashion

**Figure 2:**
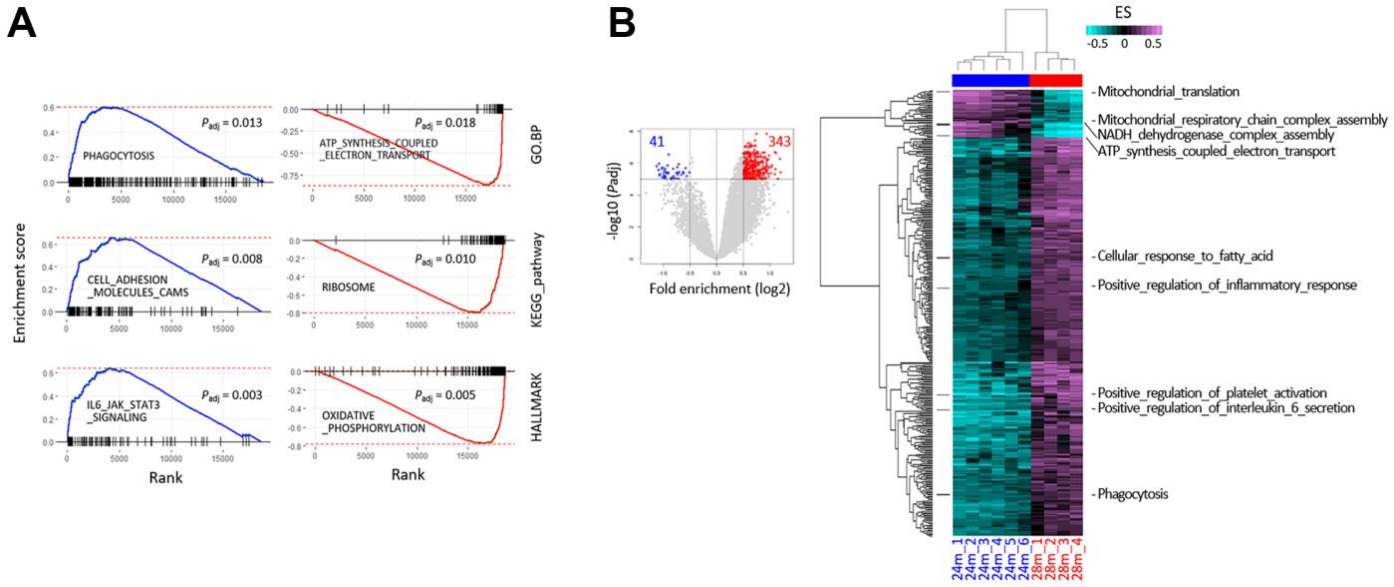
**Gene set enrichment analysis (GSEA) using RNA-sequencing data from the skeletal muscle of 24- and 28-month-old mice.** (**A**) The GSEA mountain plots representatively show significant enrichment (left) or depletion (right) of genes, for the indicated gene sets and collections. The thick blue and red lines indicate the running enrichment scores across the fold change-ranked genes (Rank), in comparison to the RNA-seq gene-level expression at 28 over 24 months. Black vertical tick marks below or above the curve indicate the location of individual target genes within the fold change-ranked gene list. Adjusted *P*-values (P_adj_, Benjamin and Hochberg-corrected enrichment statistics) are indicated. (**B**) Single-sample GSEA with gene sets showing differential enrichment in the skeletal muscle of 24- and 28-month-old mice. Using GSVA, single-sample GSEA was performed on GO.BP collection from MSigDB (v7.0; see Supplementary Figure 1 for other collections). The volcano plot shows the distribution and the number of gene sets with differential enrichment (DE; FDR < 1 × 10^–5^, log2 fold-enrichment > 0.5) between the 24 and 28 months; each dot indicates a gene set in GO.BP MSigDB collection and blue and red dots for depleted and enriched in the 28 months, respectively. The numbers in red and blue indicate the gene set numbers enriched and depleted in 28m samples, respectively. Heatmaps show differential enrichments among individual 24m and 28m samples. Samples were hierarchically clustered on the x-axis (28m, red; 24m, blue) in an unsupervised manner, and significant DE gene sets are shown on the y-axis. Black bars on the left represent the gene sets shown in Figure 4C, and the names of the gene sets are denoted on the right. Colors in the GSVA score bar indicate enrichment scores in individual samples.

We inspected the young-old and old-old muscle transcriptomes in detail, to discover genes whose expression was changed in a coordinated L-aging-specific manner, thus revealing certain cellular events disturbed during L-aging. We performed gene set enrichment analysis (GSEA) on the “HALLMARK,” “KEGG_PATHWAY,” and “GO.BP” collections using the fast pre-ranked GSEA (fGSEA) package, to interpret coordinate changes in the transcriptomes of 28m over 24m samples (Supplementary File 2). A number of gene sets were significantly enriched or depleted in the 28m transcriptomes (Figure 2A and Supplementary Figure 2). The significantly enriched genes were associated with cell adhesion, cell signaling pathways, and inflammation-related sets, whereas the significantly depleted ones were linked to oxidative phosphorylation and translation terms. Single-sample GSEA (ssGSEA) using gene set variation analysis (GSVA) was performed, which assesses separate enrichment scores (ESs) for each sample and gene set pairing, to determine the extent of coordinate gene set up- or downregulation within a sample group [19]. GSVA results demonstrated that the 28m samples were synchronized in expression levels for a variety of GO.BP gene sets (n = 384), showing a pattern opposite to that of the 24m samples (*P*_adj_ < 1 × 10^–5^ and log2 fold-enrichment > 0.5; Figure 2B and Supplementary Figure 3). Notably, genes were overrepresented in the 28m samples in the majority of the selected terms (89.3%, 343/384 terms). Our results indicated that the old-old transcriptomes are distinguishable from the young-old ones and that the transcriptomic change in L-aging might not be totally fortuitous but predictable to some degree for certain gene sets. The same gene sets selected by the bulk GSEA (Figure 2A and Supplementary Figure 3) were reproducibly chosen from the GSVA.

### Transcriptomic distance among the young, young-old, and old-old in skeletal muscle and blood cells

**Figure 3:**
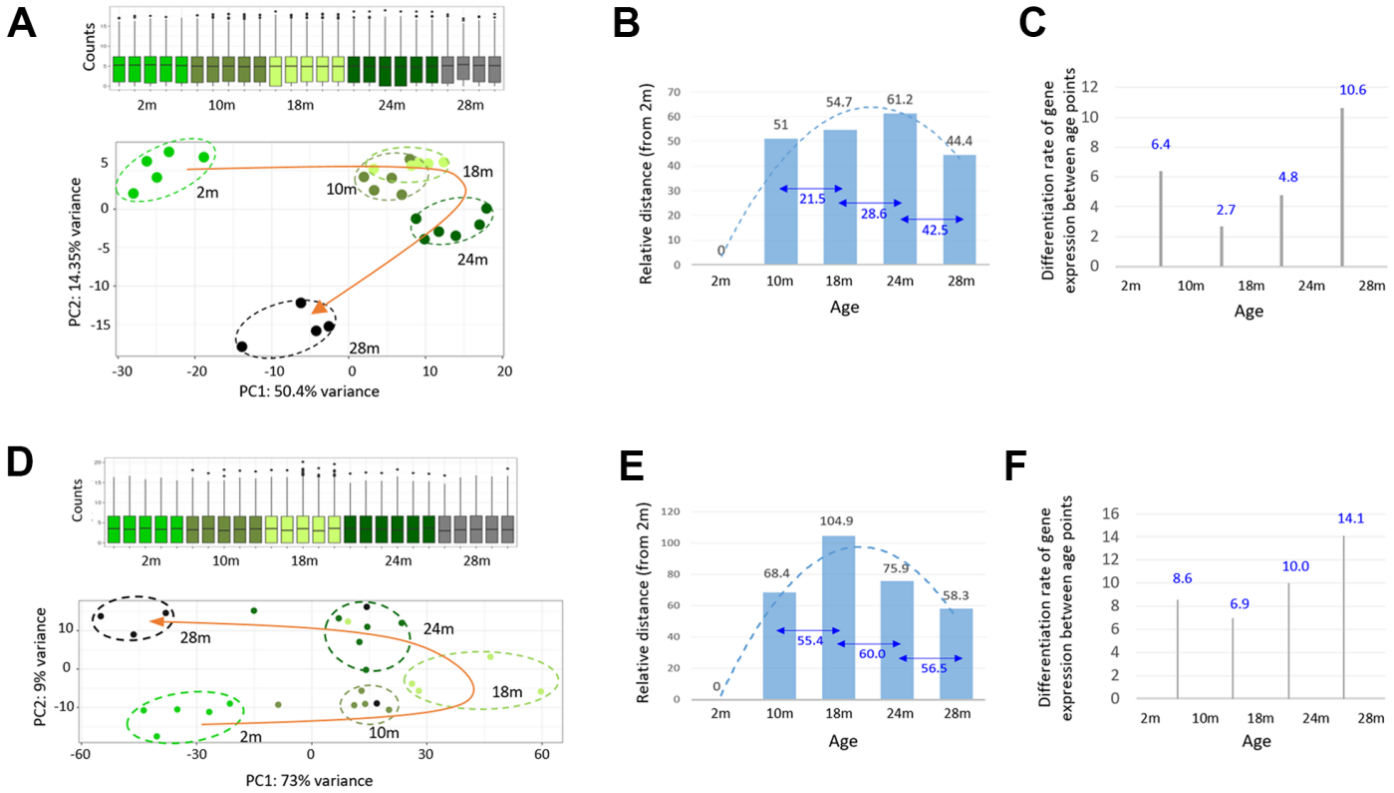
**A steep change in the transcriptome of skeletal muscle during the late phase of aging.** (**A**, **D**) Principal component analysis of the skeletal muscle (**A**) and peripheral blood mononuclear cell (PBMC); (**D**) RNA-seq data from 2-, 10-, 18-, 24-, and 28-month-old mice. Transcriptomes of different age groups are marked by different colors. The curved orange arrow connects the group mean transcriptome of each age group to show an age-associated change in the gene expression profile of the skeletal muscle. Box plots show the distribution of normalized counts. (**B**, **E**) Measurement of the group mean transcriptomic distance / variance of each age group, relative to the 2-month group (numbers in black) or among the age groups (numbers in blue). (**C**, **F**) Per-month differentiation of transcriptomes between the age groups, which divides the transcriptomic distance by the age (month) difference.

For evenly spaced chronological transcriptomes, middle-aged samples were required. Therefore, we carried out RNA-sequencing of the skeletal muscle from 10- and 18-month-old mice (n = 6). In a PCA, using the sequencing data of all age groups, we discovered that the 24m transcriptomes slightly deviated from the forward trajectory and that the 28m transcriptomes swerved completely outward; thus, both these groups disengaged from the transcriptomic path of younger age groups (Figure 3A). We measured the transcriptomic distance between the age groups based on Euclidian distance [20]. The 24m group was the furthest cluster from the 2m group, whereas the 28m group was the nearest (Figure 3B). The 10m and 18m transcriptomes clustered close to those of the 24m on the PCA plot. Interestingly, the results indicated that the old-old transcriptomes clustered closer to the young ones than the young-old ones. The monthly divergence of the transcriptome with age, calculated by dividing the transcriptomic distance between the age groups by the age difference in months, decelerated until 18 months and then accelerated as the tissue entered into the E-aging and L-aging groups (Figure 3C). The divergence rate in the L-aging period was approximately twice as high as the rate in the E-aging period (10.6 versus 4.8).

We examined peripheral blood mononuclear cells (PBMCs) to test the generality of our findings with skeletal muscle. In the PCA results, using RNA-seq data of PBMCs obtained from the same mice as that used for muscle tissue collection, the transcriptomes of different age groups demonstrated a boomerang-shaped shift with age (Figure 3D). As for transcriptomic distance, relative to the 2m group in the PBMCs, the 28m group was the nearest (Figure 3E), and the monthly divergence rate was the highest for L-aging, similar to that in the skeletal muscle (Figure 3F). Collectively, our results indicated that the transcriptomes are markedly altered during L-aging, with the trajectory steeply disengaged from the initial forward path. With regards to the gene expression profile, the old-old group clustered closer to the young one than the young-old group in both the skeletal muscle and PBMCs.

### Transcriptomic muscle signature discriminates the old-old from the young-old

**Figure 4:**
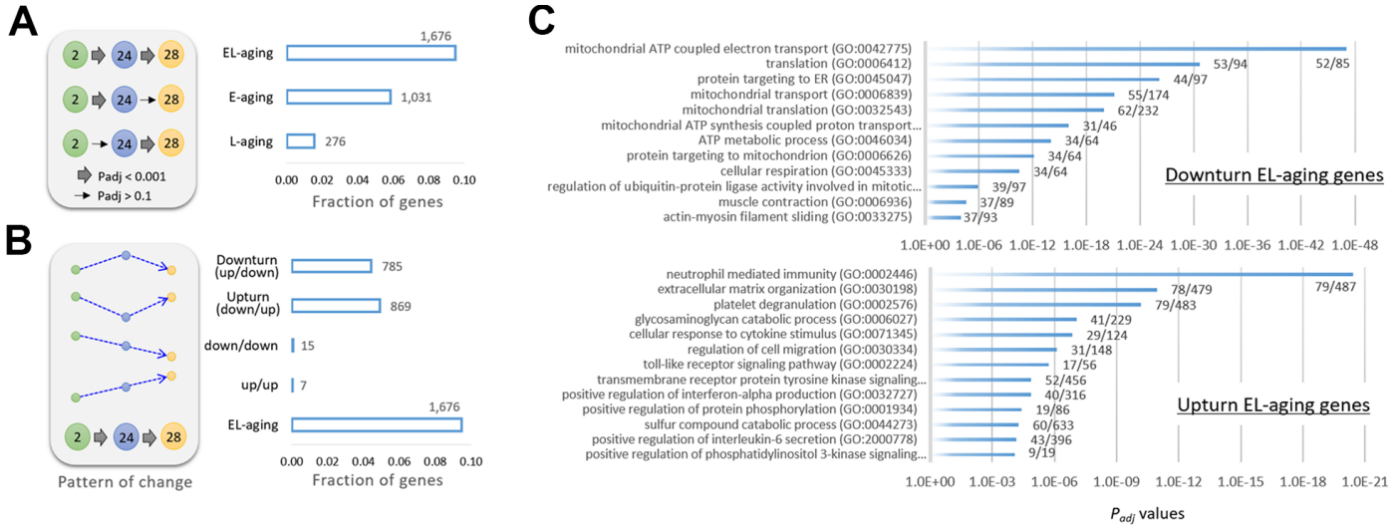
**Classification of genes by age-associated expression pattern.** (**A**) Gene categories of E-aging genes, L-aging genes, or EL-aging genes that show significant changes in expression levels during E-aging or L-aging, or in both phases (EL-aging), respectively. The thick arrow in the illustration indicates a significant change (*P*_adj_ < 0.001) among the age groups, whereas the thin arrow indicates no significance (*P*_adj_ > 0.1). (**B**) Further classification of the EL-aging genes by the pattern (dotted arrows in blue) of change in expression level with age. The number of genes in each category is indicated on the graph. (**C**) Gene ontology result using 785 downturn (top) and 869 upturn EL-aging genes (bottom). The fractional numbers indicate the number of EL-aging genes over the whole genes in the corresponding gene set.

To specify the genes that underwent transcriptional alterations during E-aging and L-aging, we classified them based on expression pattern into three types: E-aging, L-aging, or EL-aging genes. Expression levels of the first two types were significantly altered (*P*_adj_ < 0.001) in each designated period only but not in the other period (*P*_adj_ > 0.1; Supplementary File 2). In contrast, EL-aging genes showed significant changes in both the E-aging and L-aging periods in a row (Figure 4A). In total, 1,676 EL-aging genes were identified that accounted for 9.5% of all genes. Most of the EL-aging genes (except for seven and 15 genes continuously increasingly and decreasingly expressed, respectively (see [21] for the case of rat limb muscle) showed fluctuations in their expression levels, either an up-and-down (downturn; 785 genes) or a down-and-up (upturn; 869 genes) pattern at 24 and 28 months, starting at two months (Figure 4B and Supplementary File 2). Gene ontology (GO) analysis using the downturn genes, revealed that the terms were primarily related to mitochondrial function and translation involving “mitochondrial ATP coupled electron transport (GO:0042775; *P*_adj_ = 8.7 × 10^-48^),” “translation (GO:0006412; *P*_adj_ = 2.3 × 10^-31^),” and “mitochondrial transport (GO:0006839; *P*_adj_ = 7.8 × 10^-22^),” among others (Figure 4C and Supplementary File 2). GO analysis using the upturn EL-aging genes yielded terms that are related to immune reaction and cell signaling, including “neutrophil-mediated immunity (GO:0002446; *P*_adj_ = 3.9 × 10^-21^),” “extracellular matrix organization (GO:0030198; *P*_adj_ = 1.1 x 10^-11^),” and “platelet degranulation (GO:0002576; *P*_adj_ = 6.6 × 10^-11^),” to name but a few.

In total, there were 1,031 E-aging genes of which half (n = 502) was underrepresented in expression, and the other half (529) was overrepresented. Their GO analysis results are shown in Supplementary File 2. The GO output was small in number and with less significant terms (P_adj_ < 0.01) than the output from the EL-aging genes. For the L-aging genes, only a relatively small number (n = 276) were detected. GO analysis of the L-aging genes yielded no significant gene sets, even at *P*_adj_ < 0.05, on the three collections. Therefore, judging from the high statistical significance with which the gene sets were identified, the up- or downturn expression shifts in these EL-aging genes, along with the accompanying up-and-down functions in the corresponding gene sets, might be of no random pattern but have yet to be explored as consequences of the progression of aging.

**Figure 5:**
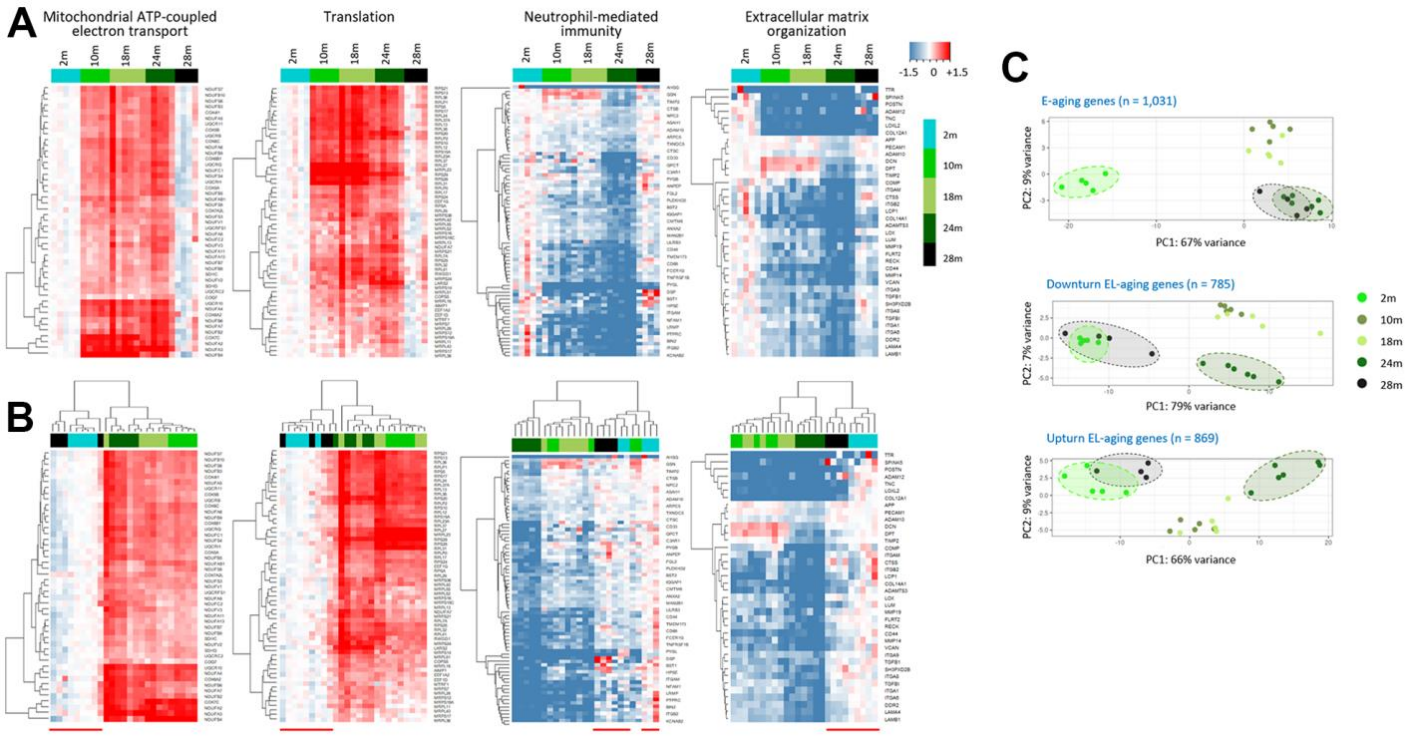
**Assimilation of the EL-aging gene expression profiles of the skeletal muscle in super elderly mice with those in the young mice.** (**A**, **B**) Heatmaps of EL-aging genes for expression levels of individual muscle samples relative to the mean level of the 2-month group. Age-associated expression changes in four representative gene sets are shown by unsupervised (**A**) or supervised hierarchical clustering (**B**) Red line below the heatmap in B indicates the cluster of 2m and 28m samples. (**C**) Comparison of transcriptomes of E-aging (top) and EL-aging genes (bottom) among the age groups. A close similarity is shown in the 24m (dark green) and 28 samples (black) for the E-aging gene group, whereas it was in the 2m (light green) and 28m samples for the EL-aging gene group and their down- and upturn subsets.

Through hierarchical clustering, we examined changes in the expression levels of EL-aging genes in the identified gene sets. As shown in the heatmaps of the four representative gene sets (Figure 4C), the down- or upturn pattern of age-associated changes was clearly shown by supervised clustering (Figure 5A). In the period spanning the middle age and E-aging (10 to 24 months), the genes either maintained their expression levels relatively constant (“mitochondrial ATP coupled electron transport” and “translation” sets) or showed a gradual decline (“neutrophil-mediated immunity” and “extracellular matrix organization” sets); however, no abrupt change in expression was found among the EL-aging genes in this period. Unsupervised clustering data showed that the 28m samples were closely associated with the 2m samples in all the gene sets (Figure 5B). From the analysis of public mouse muscle transcriptome data [22], we confirmed a similar downturn change in expression levels of “mitochondrial ATP coupled electron transport” gene set with age (Supplementary Figure 4). Since these public transcriptome data came from male mice, we assume no difference in the age-linked expression pattern of EL-aging genes between the sexes. To see if there was an age-related change in the mitochondrial copy number in the skeletal muscle, we determined the copy number ratio of the mitochondrial 16S gene sequence relative to a nucleus-encoded, single-copy gene sequence (HK2) [23] in the tissue DNAs from 2, 10, 18, 24, and 28 month old mice that were the same batches of muscle tissues that were used for the transcriptome analysis above. The result indicated that the copy number of mitochondria in the skeletal muscle showed a downturn pattern of change with age (Supplementary Figure 5), and that the number of mitochondria strongly correlated with the mean expression levels (r = 0.892; Pearson correlation) of “mitochondrial ATP coupled electron transport” genes. Meanwhile, an assimilation was demonstrated in the PCA plots for the EL-aging genes of the 28m group expression profiles, relative to those of the 2m group. For the 1,031 E-aging genes, the 28m samples overlapped with the 24m samples, whereas they overlapped with the 2m samples for the 785 downturn and 869 upturn EL-aging genes (Figure 5C). This indicated that the transcriptional similarity of old-old muscles with that of young muscles is not restricted to certain gene sets but appears across all the EL-aging genes.

## DISCUSSION

In summary, we observed a comprehensive change in the transcriptome of skeletal muscle during L-aging. The transcriptomes of old-old samples were markedly altered, exhibiting a drastic change in the forward path manifested by the transcriptomes of the younger age groups. Many genes were significantly changed, and of them, the EL-aging genes demonstrated fluctuation in their expression levels with a successive change during the E-aging and L-aging period. However, these changes among old-old samples did not seem to be random but rather synchronic in a variety of gene sets. For example, increasingly expressed EL-aging genes in the L-aging group were significantly enriched in the immunity- and inflammation-related gene sets, whereas decreasingly expressed genes were depleted in the mitochondrial function and translation terms.

We assume that during the E-aging, the EL-aging genes are either in highly expressed or tightly repressed states and multi-layered regulatory systems struggle for transcriptional homeostasis at the expense of cellular energy. During the L-aging, as cellular energy and resources become limited, cells and their transcriptional regulatory systems give way to being decompensated throughout the genome, as evidenced by the upturn and downturn shifts of expression. As such dysregulations over the genome continue unchecked and wide-spread, it eventually results in systemic aging. Likewise, the tension-releasing shift can passively occur in aged, decompensated cells, or there may exist an unknown factor that triggers such changes yet to be identified. At the molecular level, within a cell, genetic and epigenetic regulatory devices that act on the gene sets (Figure 4C) involving EL-aging genes have hitherto managed to homeostatically control the transcriptional milieu over the genes. These devices may break down by increasing stress and tension elicited with aging, leading to the awry expression of genes. Some early EL-aging genes, when altered in expression levels, may accelerate cells to transit to the late phase of aging and further transcriptionally alter the other downstream EL-aging genes. If we could identify these leading EL-aging genes and determine how to keep them safe and unharmed from causes and results of aging, we could delay the oncoming L-aging and prolong the slow E-aging. This can undoubtedly be the genuine way for healthy aging.

There was a substantial change in the gene expression profile of skeletal muscle during L-aging, as visualized by the large swerving trajectory of old-old transcriptomes in the PCA results (Figure 3A). The EL-aging genes might play an important part in this process, considering the expression profile of these genes in old-old samples that largely overlapped with those in young samples (Figure 5C). This “reversion-toward-the-young” event is distinguished from the “regression-toward-the-mean” case often manifested in transcriptomic analysis of aging samples, in that, the latter illustrates antithetical directions of changes occurring between high and low transcribed genes, which results in reducing the gene-to-gene disparities in transcription with age [24]. Regarding an analytical method, whereas the “regression-toward-the-mean” event is obtained from a simple two-point comparison of individual genes with resultant fold-change swaying on the reference time point used, the “reversion-toward-the-young” event could be acquired from a chronological tracing of gene expression levels at multiple time points, thus unwavering and more faithful. In terms of the fraction of (EL-aging) genes showing the “reversion-toward-the-young” change, 1,676 genes were output after the *P*_adj_ = 0.001 cut-off. When the cut-off was lowered to *P*_adj_ = 0.05, the number increased to 3,996, which is 22.6% of all the genes, a large fraction enough to call the movement a global pattern. We previously observed this “reversion-toward-the-young” pattern of expression change, among genomic retroelements in mouse skeletal muscle [16]. Most subfamilies of LINE1s and LTRs showed an upturn change in expression levels (apart from the statistical significance of the changes); their initial high-level expression at two months was diminished at 20 months and then increased again at 28 months. Given the scattered presence of retroelement sequences over whole chromosomes and the large fraction of EL-aging genes, we suggest that the age-linked up- and downturn changes in expression are genome-wide trends in aged cells and tissues.

Of course the expression “the reversion-toward-the-young” does not mean the physical rejuvenation of skeletal muscles; it just portrays the assimilation of 28m muscle to 2m muscle regarding gene expression profile, which we believe is important in that the expressions of certain sets of genes with specific cellular functions are not individually randomly changed but appear to be coordinately regulated during the late phase of aging. There might be an argument that since mature adult mice are usually defined as 3–6 months old, any differences between 2 months and older time points are confounded by these maturational changes and cannot be ascribed solely to aging. Our result indeed showed a big shift in the expression levels of ‘Oxidative phosphorylation’ and ‘Translation’ set genes between 2m and 10m muscles (Figure 5A), favoring the notion of muscle immaturity in 2m samples. It thus recommends to be careful of choosing sample age for comparison of skeletal muscle and to re-consider the use of muscle sample from immature, or mature but still young, mice as a reference. As we made the point clear above, however, we focused on aging events during the late phase of aging and were particularly interested in changes between young-old and old-old. We first found abrupt changes in gene expression levels during the L-aging, and next observed the resulting profiles of the changes overall resembling those of 2m muscle samples. Hence, our results put no interpretative confusion or ambiguous boundary between aging and maturation changes of sample tissues.

Most age-related diseases are very complex and their etiologies cannot simply be assigned to specific genes; hence, it is hard to match the transcriptional changes of EL-aging genes with certain disorders in the elderly. Nevertheless, the decreasing mitochondrial function and translation (related to the downturn genes) and the increasing inflammation (related to the upturn genes) in the old-old muscle samples accord with the causes of sarcopenia [11]. Sarcopenia is a progressive skeletal muscle disorder involving the accelerated loss of muscle mass and function and occurs commonly as an age-related process in older people, influenced not only by contemporaneous risk factors, but also by genetic and lifestyle factors operating across the life course [25]. In attempt to search for genes associated with sarcopenia and to understand the underlying mechanism driving sarcopenia, several studies investigated changes in muscle gene expression with age. Kang et al. recently identified 15 DEGs from skeletal muscle of sarcopenia patients [26] and, surprisingly, the majority (10/15) of them corresponded with our mouse DEGs (28m vs. 24m; *P*_adj_ <0.05): five DEGs overrepresented (*Runx1, Bcl3, Acsl5, Ngfr*, and *Chi3l1*) and five underrepresented (*Ndufb5, Tcap, Slc253a, Cyc1*, and *Acat1*) in old-old samples. It indicates that the muscle biomarkers identified from human sarcopenia patients are similarly significantly altered in mouse skeletal muscle during L-aging, suggesting that both species share DEGs to a degree in skeletal muscle aging. In addition, from analysis of skeletal muscle transcriptomes, Mahmassani et al. identified 30 DEGs altered by bed rest in old subjects that correlated with change in leg lean mass [27]. Of the 22 DEGs (eight genes were missed in our transcriptome data), 12 genes were also significantly changed in this study during L-aging (*Arsb, Kctd10, Coq10a, Sort1, Fam96a, Gas1, Thbs4, Avpr1a, Col22a1, Retsat, Ankrd50,* and *Xpc*; *P*_adj_ <0.02). This comparative study supports the notion that those genes we identified as DEGs from the comparison of young-old and old-old samples are implicated in muscle weakening and, possibly, the associated disorders in humans. Meanwhile, sarcopenia is associated with type 2 diabetes in the elderly [28] and, in a re-analysis of public microarray data from human skeletal muscle biopsies, Su et al. found that three age-related genes were associated with type 2 diabetes [29]; two of them, *Cd163* and *Gadd45a* (the remained *Znf415* was missed in our study) were classified as the L-aging (*P*_adj_ = 1.0 x 10^-5^) and E-aging genes (8.8 x 10^-11^), respectively. In conclusion, what the exact consequences of altered expressions of all these DEGs that were shared in humans and mice are currently unknown. Nonetheless, our transcriptomic signature for the old-old muscle and those failure-prone gene sets acquired by the impact assessment of the late aging phase can be regarded as molecular and physiological traits by which the old-old are distinguishable from the young-old. Therefore, we hope that the ‘old-old’ muscle transcriptome may serve as a point of reference for some predisposed disorders in the super-elderly.

Previous studies showed different rate of muscle loss between sexes during aging. They recognized malnutrition in females and higher serum myostatin in males as different risk factors for sex-specific difference of muscle aging in humans [30, 31]. We here examined muscle aging in female mice only. So, we cannot exclude the possibility that the males may differ from the females in late phase of aging. Although the analysis of public muscle transcriptome data from male mice suggested no sexual difference in the expression pattern of “mitochondrial ATP coupled electron transport” gene set in life (Supplementary Figure 4), it should be noted that mice (and rat also) have a different survival rate depending on sex and strains (up to four-month difference at 50% survival age; Strain Survival Information, https://www.nia.nih.gov), which suggests a caution in simple comparison by age between sexes and requires an additional correction for the large gender gap in survival.

In conclusion, we examined the transcriptomes of skeletal muscle obtained from 24- and 28-month-old mice as the young-old and old-old groups, respectively, along with 2-, 10-, and 18-month-old mice representing young and midlife controls. Through the sub-classification of the old into the young-old and the old-old, we were able to observe a global change in the muscle transcriptome during the late phase of aging, and the changes among the old-old appeared rather synchronic in a variety of functional gene sets. Our results provide a transcriptomic muscle signature that distinguishes old-old mice from young-old mice, which we hope would help to understand skeletal muscle aging in late life and to methodically examine muscle disorders in the elderly with the impact assessment of the late aging phase provided in this study.

## MATERIALS AND METHODS

### Ethics statement

This study was carried out in strict accordance with the recommendations in the Guide for the Care and Use of Laboratory Animals of the National Livestock Research Institute of Korea. The protocol was approved by the Committee on the Ethics of Animal Experiments of the Korea Research Institute of Bioscience and Biotechnology.

### Isolation of skeletal muscle and peripheral blood mononuclear cells (PBMCs)

C57BL/6J female mice at 2, 10, 18, 24, and 28 months of age (four to six in number for each age group) were purchased from LARC (KRIBB) immediately before sacrifice. During organ harvest surgery, neither signs of cachexia nor tumors were found in older mice as well as in younger mice. To obtain skeletal muscle, mice were sacrificed and skeletal muscle in hind limbs were surgically removed and minced. The whole lot of minced tissues were quickly frozen in liquid nitrogen, ground to powder using a mortar and a pestle, and stored in small volumes in -80° C for later use. The powdered tissues were further homogenized using a Biomasher II (DWK Life Sciences) in tissue lysis buffer (ATL buffer; Qiagen) as described before [24]. For collection of PBMCs, the same mice as that used for muscle tissue collection were used. Whole blood was drawn from mouse heart using one ml syringe and immediately mixed with 2 mg EDTA (pH 7.4) per ml of blood to prevent coagulation [32]. The EDTA-treated whole blood was incubated with ten times volume of ACK Lysing Buffer (Thermo) at RT for 10 min to remove red blood cells. The mixture was centrifuged and supernatants were removed. The pellet was resuspended in 5 ml ACK Lysing Buffer to completely remove the residual red blood cells, and PMBCs were collected by additional centrifugation. PBMCs were washed with 1 ml phosphate-buffered saline (PBS) and aliquoted in 1.5 ml tubes to be stored in -80° C.

### RNA-seq library construction

Total RNA was extracted from 30 mg of the muscle powder lysed in 200 μl of TRIzol Reagent (Thermo). Poly-A tailed RNAs were isolated from 1 μg of total RNA using Dynabeads mRNA DIRECT kit (Thermo) according to the manufacture’s recommendation. The purified Poly-A tailed mRNAs were treated with DNase I (Sigma) for complete elimination of residual genomic DNAs (gDNA) for 30 min at 37° C prior to RNA-seq library generation. Next, RNA-seq libraries were generated by NEBNext Ultra RNA Library Prep Kit for Illumina (NEB) as described in the provider’s protocol. Briefly, the gDNA-free mRNAs were incubated at 94° C for 15 min for fragmentation. First strand cDNA was synthesized with fragmented RNAs using ProtoScript II Reverse Transcriptase and their second strands were synthesized using Second Strand Synthesis Enzyme Mix in the kit before purification. After the end repair of the double-stranded DNAs using NEBNext End Prep Enzyme Mix in the condition of 20° C for 30 min and 65° C for 30 min, the products were incubated with NEBNext Adaptor and Blunt/TA Ligase Master Mix (NEB) at 20° C for 15 min. The resulting ligates were enriched by 12 - 15 cycles of PCR by universal and index primers using 2 x Phusion High-Fidelity PCR Master Mix with HF Buffer (Thermo). Enriched RNA-seq libraries were quantified using NEBNext Library Quant Kit for Illumina (NEB), pooled them together by their quantities, and then sequenced by Illumina HiSeqX system (2 x 100bp).

### RNA-seq data analysis

Raw sequencing reads were preprocessed to remove adapter sequences and low quality bases using ‘Trim_galore v0.6.0’ (https://www.bioinformatics.babraham.ac.uk/projects/trim_galore/). Next, the reads were aligned on the reference genome (mm10) using ‘STAR v2.7.0’ [33], and the resulting SAM files were converted to sorted BAM files using ‘samtools v1.9’ [34] with ‘-q 1’. Raw gene expression levels were computed by ‘htseq-count v0.11.1’ [35], and an expression matrix combined all sample data was generated by a home-brew bash script.

The raw expression dataset was normalized using ‘DESeq2 v1.30.0’ [36], and gene expression levels between age groups were calculated to identify differentially expressed genes (DEGs, fold-change > 2, adjusted *P* value < 0.001 if not indicated otherwise). For principal component analysis (PCA), the expression dataset was normalized by variance stabilizing transformation, and samples were plotted on a 2D PC plain (PC1 x PC2) by ‘pcaplot’ function (ntop = 5,000) in DESeq2 for visualization. Transcriptomic divergence or distance between age groups, which is based on Euclidean distance, was computed by ‘dist’ function using whole genes in R. All plots were generated using ‘ggplot2’ and ‘heatmap2’ functions in R or MS EXCEL. R codes for DESeq2 analysis are available in <LINK>.

### Differential expression analysis and GSEA analysis

For gene set enrichment analysis (GSEA), gene collections for ‘HALLMARK’, ‘KEGG pathways’, and ‘GO Biological Process’ from MSigDB v7.0 [37, 38] were obtained, and gene sets enriched in either 24- or 28-months-of-age group were identified using fGSEA [39]. In addition to the fGSEA that was generated based on group means, single-sample GSEA (ssGSEA) was performed using gene set variation analysis (GSVA) [40], a R package for gene set variation analysis among individual samples. First, the normalized count data from ‘DESeq2’ were converted to an input matrix for ‘GSVA’ by a home-brew R code, and enrichment scores (ES) for individual samples were calculated by the ‘gsva’ function with ‘method=gsva’. Then, using ‘limma’ [41], a R package for differential expression analysis, gene sets with significantly altered activations (FDR < 1 x 10^-5^) were identified, and the results were visualized on volcano plots and heatmaps in R [42].

### RT-PCR and calculation of mitochondrial DNA copy number

Total RNAs were separately extracted from limb muscle tissues of 24-month- (n=6) and 28-month-old (n=4) mice as described above and pooled by age group. Reverse transcription was performed by incubating 1 μg of DNase I-pretreated RNA with Superscript III enzyme (Invitrogen), 20 μM oligo dT primers (Invitrogen), and 50 ng random hexamers (Invitrogen) at 50° C for 1 h. Ten ng of the synthesized cDNA was used for a real-time quantitative PCR (QuantStudio3 Real-Time PCR system, ABI) with the specific primers for individual retroelement subfamilies (Supplementary Figure 6). PCR was performed with a following program; 10 minutes of pre-denaturation at 95° C followed by 40 cycles of 95° C / 15 sec and 60° C / 1 min. Finally, relative expression level of each gene to *Gapdh* was calculated using QuantStudio Design and Analysis Software (Thermo).

For estimating the ratio of mitochondrial DNA relative to nuclear DNA, mitochondrial 16S rRNA gene and nuclear-encoded hexokinase 2 (HK2) gene were selected [23]. Using mouse skeletal muscle genomic DNA at different months of age as template and 2X Power SYBR Green PCR Master Mix (ABI), a quantitative real-time PCR was performed in QuantStudio 3 Real-Time PCR System (ABI). Primers used were 5’-CCGCAAGGGAAAGATGAAAGAC-3’ and 5’-TCGTTTGGTTTCGGGGTTTC-3’ for 16S rRNA gene sequence, and 5’-GCCAGCCTCTCCTGATTTTAGTGT-3’ and 5’-GGGAACACAAAAGACCTCTTCTGG-3’ for HK2 gene sequence [23]. PCR condition was set as 45 cycles of 95° C / 10 sec, 60° C / 10 sec, and 72° C / 20 sec. The ratio of mitochondrial DNA relative to nuclear DNA was calculated by the classical ΔΔCT method used for qPCR analysis.

### Data availability statement

The data that support the findings of this study are openly available in Gene Expression Omnibus (GEO) with accession number of GSE.

## Supplementary Material

Supplementary Figures

Supplementary File 1

Supplementary File 2
