# Supplementary material for: Different phases of aging in mouse old skeletal muscle
Source: Aging (Albany NY). 2022 Jan 11;14(1):143–60. doi: 10.18632/aging.203812 (PMC8791220; doi:10.18632/aging.203812)
Supplement: Supplementary Figures [file aging-14-203812-s001.pdf]

SUPPLEMENTARY FIGURES

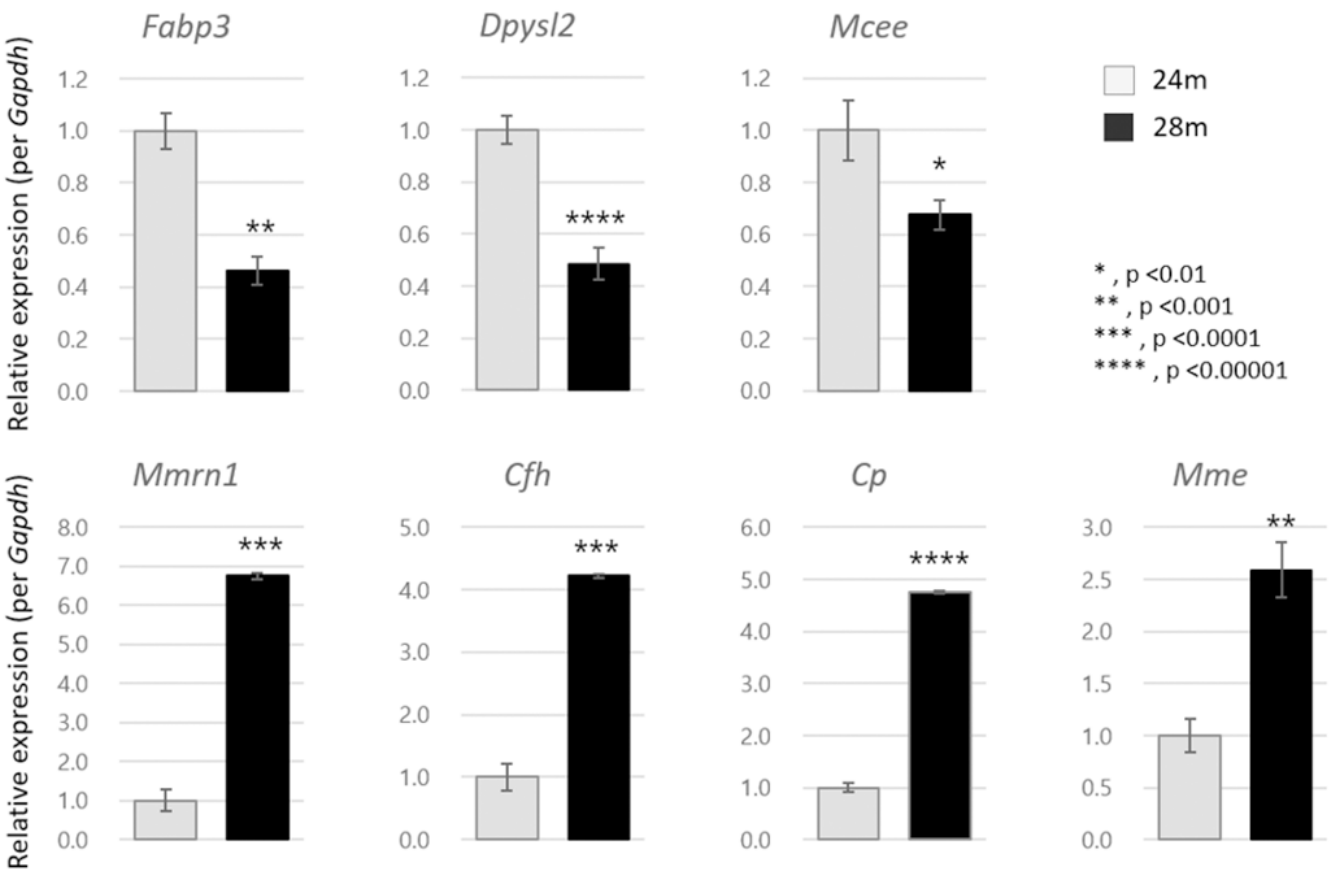

Supplementary Figure 1. Quantitative real-time- PCR for validation of differential expression of genes between the young-old (24m) and old-old (28m) muscle samples. Asterisks indicate significant different in expression levels. Error bars, standard deviations. *Gapdh* transcript level was used as an internal control.

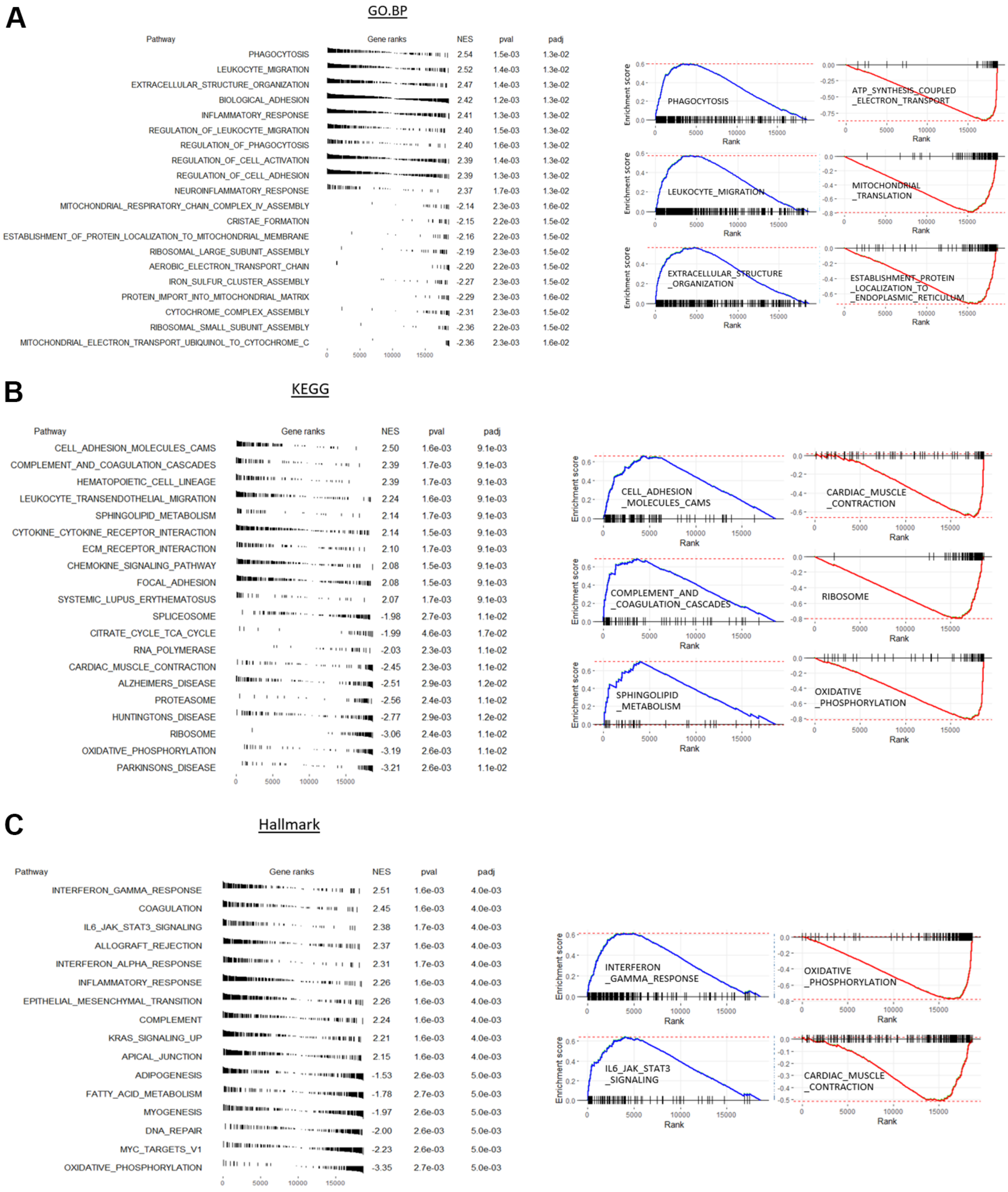

Supplementary Figure 2. Fast pre-ranked gene set enrichment analysis (fG5EA) result on GO. BP (Gene ontology-Biological process; (**A**), KEGG_pathway (**B**), and Hallmark (**C**) collections using RNA-seq data obtained from the mouse skeletal muscle at 24 and 28 months of age. Left panel in (**A**–**C**) shows a tabulated result using plotGseaTable function in R, and right panel the GSEA mountain plot which representatively showing a significant enrichment (left) or depletion (right) of genes for indicated gene sets and collections. Thick blue and red lines indicate the running enrichment scores across the fold change-ranked genes (Rank) in comparison between the RNA-seq gene-level expression of 28 months over 24 months. Black vertical tick marks below or above the curve indicate the location of individual target genes within the fold change-ranked gene list. NES, normalized enrichment score.

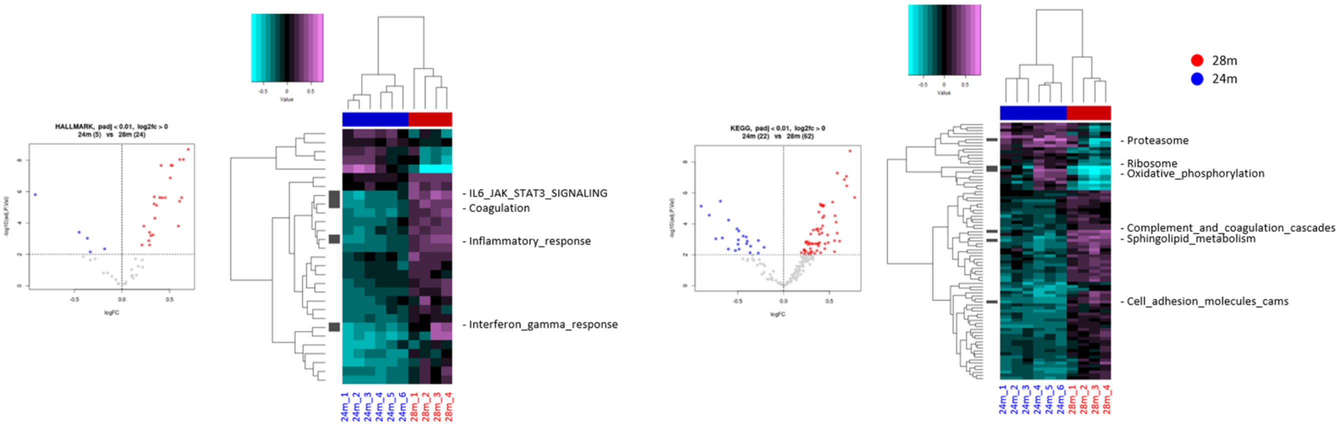

Supplementary Figure 3. Single sample GSEA with gene sets showing differential enrichments in the skeletal muscle of 24- and 28-month-old mice. Using GSVA, single sample GSEA was performed on Hallmark (left) and KEGG_pathway (right) collection. Volcano plot shows the distribution and the number of gene sets with differential enrichments (DE; *FDR* < 1 x 10^{-5}) between the 24 months and 28 months; each dot indicates a gene set in each collection, blue and red dots for depleted and enriched in the 28 months, respectively. Heatmaps show the differential enrichments among individual 24m and 28m samples. Samples are hierarchically clustered on x-axis (28m, red; 24m, blue) in a unsupervised way, and significant DE gene sets are shown on y-axis. Black bars on the left represent the gene sets shown in Figure 4C, and the names of the gene sets are denoted on the right. Colors in GSVA score bar indicate enrichment scores in individual samples.

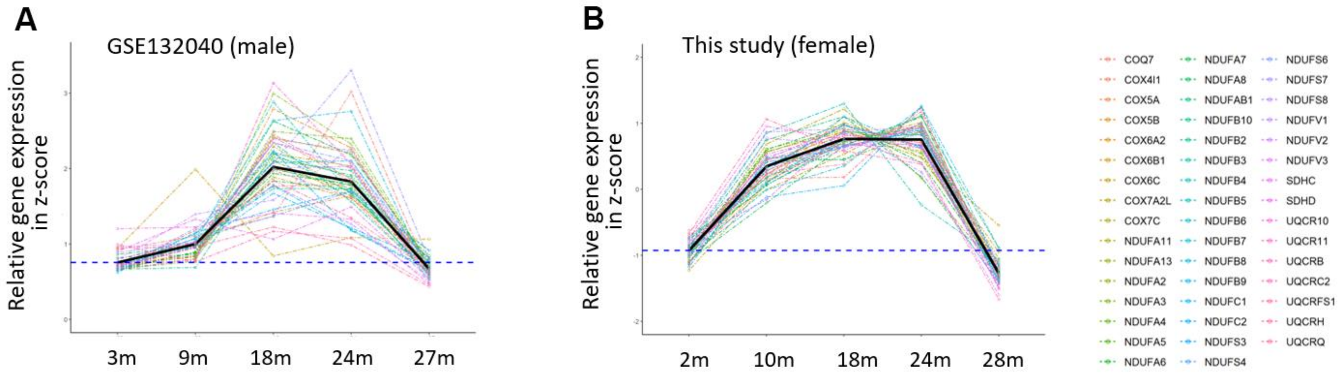

Supplementary Figure 4. Expression patterns of "mitochondrial ATP coupled electron transport" genes with age in skeletal muscle. Gene expression patterns in the public mouse RNA-seq data (GSE132040, **A**) obtained from male limbs were compared with those in our RNA-seq data (**B**; female). Black line indicates the mean expression level of the genes and the dotted blue line a reference point (z-score of 3m (**A**) or 2m (**B**) samples).

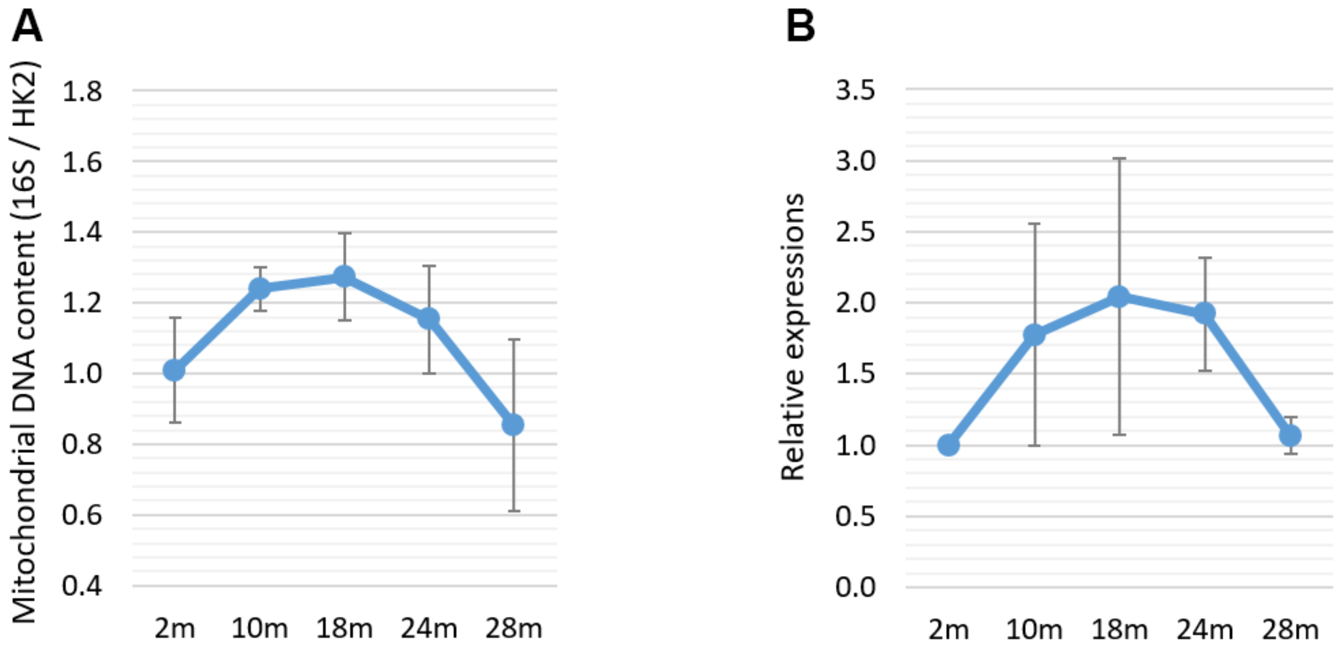

Supplementary Figure 5. Mitochondrial DNA (mtDNA) copy number was determined in skeletal muscle at different ages (**A**) and its age-linked change was compared with the change of expression levels of "mitochondrial ATP coupled electron transport" genes (**B**). The mtDNA coy number per nuclear DNA copy number was calculated using quantitative real-time PCR for the 16S mitochondrial gene and hexokinase-2 (HK2) nuclear encoded gene. Error bar indicates standard deviation.

Gene ID	Forward primer (5' to 3')	Reverse primers (5' to 3')	size (bp)	Reference
Fabp3	ACGGGCAGGAGACAACACTA	CCACACTGCCATGAGTGAGA	75	NM_010174.2
Mme	AATTCAGCCAAAGCAAGCAG	TGATTTCGGCCTGAGGAATA	109	NM_001357335.1
Mmrn1	ACCAGGCTGAAAGTCACACC	CTTCCTCTCCTGTTGGCTCA	119	NM_001163507.1
Mcee	GAACTGCTTCATCCACTGGG	AGCTGCACTGATGTTGTCCA	111	NM_028626.2
Cp	GTGAAGAGACGAGCCGAAGA	TATGGCCTGGTTGCCATATT	110	NM_007752.3
Dpysl2	ATCTCTGCCAAGACACACAACA	ACATGAAGTGTGCCGTCCTC	125	NM_009955.3
Cfh	CAAATGAATGGCTCAGACACTG	ATTTGGCACATGTGGTGGA	102	NM_009888.3

Supplementary Figure 6. Primer sequence information used in quantitative real-time PCR.
